# Selector genes display tumor cooperation and inhibition in *Drosophila* epithelium in a developmental context-dependent manner

**DOI:** 10.1242/bio.027821

**Published:** 2017-11-15

**Authors:** Ram Prakash Gupta, Anjali Bajpai, Pradip Sinha

**Affiliations:** Department of Biological Sciences and Bioengineering, Indian Institute of Technology Kanpur, Kanpur 208016, India

**Keywords:** Selector genes, Cell fate, Cooperative tumorigenesis, *Drosophila*

## Abstract

During animal development, selector genes determine identities of body segments and those of individual organs. Selector genes are also misexpressed in cancers, although their contributions to tumor progression per se remain poorly understood. Using a model of cooperative tumorigenesis, we show that gain of selector genes results in tumor cooperation, but in only select developmental domains of the wing, haltere and eye-antennal imaginal discs of *Drosophila* larva. Thus, the field selector, Eyeless (Ey), and the segment selector, Ultrabithorax (Ubx), readily cooperate to bring about neoplastic transformation of cells displaying somatic loss of the tumor suppressor, Lgl, but in only those developmental domains that express the homeo-box protein, Homothorax (Hth), and/or the Zinc-finger protein, Teashirt (Tsh). In non-Hth/Tsh-expressing domains of these imaginal discs, however, gain of Ey in *lgl^−^* somatic clones induces neoplastic transformation in the distal wing disc and haltere, but not in the eye imaginal disc. Likewise, gain of *Ubx* in *lgl^−^* somatic clones induces transformation in the eye imaginal disc but not in its endogenous domain, namely, the haltere imaginal disc. Our results reveal that selector genes could behave as tumor drivers or inhibitors depending on the tissue contexts of their gains.

## Abstract (precis)

**Summary:** This study examines the consequences of gain of cell fate selector genes in oncogenically targeted cells and reveals their two contrasting developmental context-dependent outcomes: namely, tumor cooperation and tumor inhibition.

## INTRODUCTION

During animal development, hierarchical order of expression of selector genes, which are broadly classified into segment-, field- and cell fate-specific selectors (for review, see Akam, 1998; Mann and Carroll, 2002), regulate the specialization of individual body segments as well as those of the organs developing therein. Misexpression of these selector genes, on the other hand, results in transdifferentiation (transdetermination) of one body part into another (Maves and Schubiger, 2003). For instance, gain of the homeotic selector for the third thoracic segment (T3) selector, Ultrabithorax (Ubx) (Lewis, 1978), in the second thoracic segment (T2) results in T2-to-T3 transformation, marked by wing-to-haltere homeotic changes. Conversely, T3-to-T2 homeotic transformation occurs when Ubx is lost in the T3 segment (Lewis, 1978; Weatherbee et al., 1998). Further, within each segment, fates of individual organs are determined by designated field selectors. Early during development, for instance, in the epithelia of larval imaginal disc, the primordia of future adult appendages, such as the eye, haltere or the wing, display expression of the homeo-domain-containing transcription factor, Homothorax (Hth), and the zinc finger transcription factor, Teashirt (Tsh), which define their developmental ground state (Azpiazu and Morata, 2000; Bessa et al., 2002; Zirin and Mann, 2004). Field-specific selectors are then expressed, carving out the zone of future organ primordia, such as the wing, haltere or eye. Thus, expression of the Vestigial (Vg) (Kim et al., 1996; Williams et al., 1991) and Eyeless (Ey), a homolog of human Pax6 (Quiring et al., 1994), field selectors in the wing and in the eye imaginal discs suppress expression of Tsh and Hth, thereby marking the developmental domains of the future wing and the eye, respectively (Azpiazu and Morata, 2000; Bessa et al., 2002; Lopes and Casares, 2010; Peng et al., 2009; Wu and Cohen, 2002; Zirin and Mann, 2004). Ectopic expression of the Vg and Ey field selectors, on the other hand, result in development of ectopic wings (Kim et al., 1996) and eyes (Halder et al., 1995), respectively.

Acquisition of characteristic cell fate, as in the Vg-expressing distal wing imaginal discs, is also accompanied by heightened levels of tissue surveillance, resulting in rapid elimination of somatic clones that are incongruent with their neighbors with respect to their state of cell signaling (Adachi-Yamada and O'Connor, 2002; Vincent et al., 2011), cell fitness (Moreno et al., 2002) or cytoarchitecture (Tamori et al., 2016). When somatic clones display altered cell fate, these sort out from the rest of epithelium as benign outgrowths or polyps (Bielmeier et al., 2016).

Cancer cells often display reversal to a progenitor-like cell state (Friedmann-Morvinski and Verma, 2014) or exhibit switch between two distinct cell states, such as luminal and basal, as seen in prostate (Goldstein et al., 2010) and breast (Chaffer et al., 2011; Molyneux et al., 2010) cancers. Interestingly, *Drosophila* models of carcinogenesis also recapitulate fate reversals (Janic et al., 2010; Khan et al., 2013; Turkel et al., 2013). Further, solid cancers of diverse genetic and tissue origins are seen to misexpress homeotic selectors (Abate-Shen, 2002; Samuel and Naora, 2005; Shah and Sukumar, 2010). For instance, HoxC8, the mammalian equivalent of the *Drosophila* Ubx, is upregulated in human prostate cancer (Waltregny et al., 2002), while normal prostate development involves expression of other Hox genes, namely, Hox A9-11, A13, B13 and D13 (for review, see Javed and Langley, 2014). Likewise, Pax6, a homolog of mouse *small eye* (*say*) and *Drosophila ey* (Quiring et al., 1994; van Heyningen and Williamson, 2002), is upregulated in breast (Xia et al., 2015) and pancreatic (Mascarenhas et al., 2009) cancers. In this regard, it may be further noted that cancer cells harbor many mutations, of which only a few can be designated as driver mutations based on their definitive contribution to tumor progression (for review, see Stratton et al., 2009; Vogelstein et al., 2013), while the rest, which are inconsequential to tumor growth, are referred to as passenger mutations. It is also conceivable that some mutations could even reinforce the state of cell fate commitment or differentiation in an oncogenically targeted cell and prevent its tumor progression. In such a scenario, these mutations could be referred to as tumor inhibitors (Stratton et al., 2009); however, these are likely to go undetected in the absence of selection. In the context of the deregulation of selector genes in multiple cancers, notwithstanding their abundance (Bhagwat and Vakoc, 2015; Bhatlekar et al., 2014; Shah and Sukumar, 2010), it is presently uncertain if these play the roles of tumor drivers, passengers or inhibitors.

Exploration of the essential roles of cell fate selector genes during carcinogenesis can be made in genetically tractable model organisms such as the fruit fly, *Drosophila*. The *Drosophila* model of cooperative tumorigenesis (Brumby and Richardson, 2003; Khan et al., 2013; Pagliarini and Xu, 2003) is particularly amenable to probe such essential cancer mechanisms. Here, using the MARCM technique (Lee and Luo, 2001), which allows genetic loss of a tumor suppressor with accompanying gain of expression of a chosen fate selector, we have examined the developmental contexts where gain of a chosen segment- or field-selector display tumor progression or otherwise. In this test model, cells that display loss of the tumor suppressor, Lgl (Gateff, 1978), are eliminated by tissue surveillance mechanisms (Agrawal et al., 1995; Froldi et al., 2010; Igaki et al., 2009; Khan et al., 2013) in the larval imaginal discs. We gained expression of individual selector genes in the *lgl^−^* clones by MARCM technique and tested their ability to rescue these mutant cells from elimination and induce their neoplastic transformation. Our findings reveal developmental underpinnings of both tumor cooperation and inhibition by selector genes.

## RESULTS

### Fate specification of body segments and appendages in *Drosophila*

**Fig. 1.**
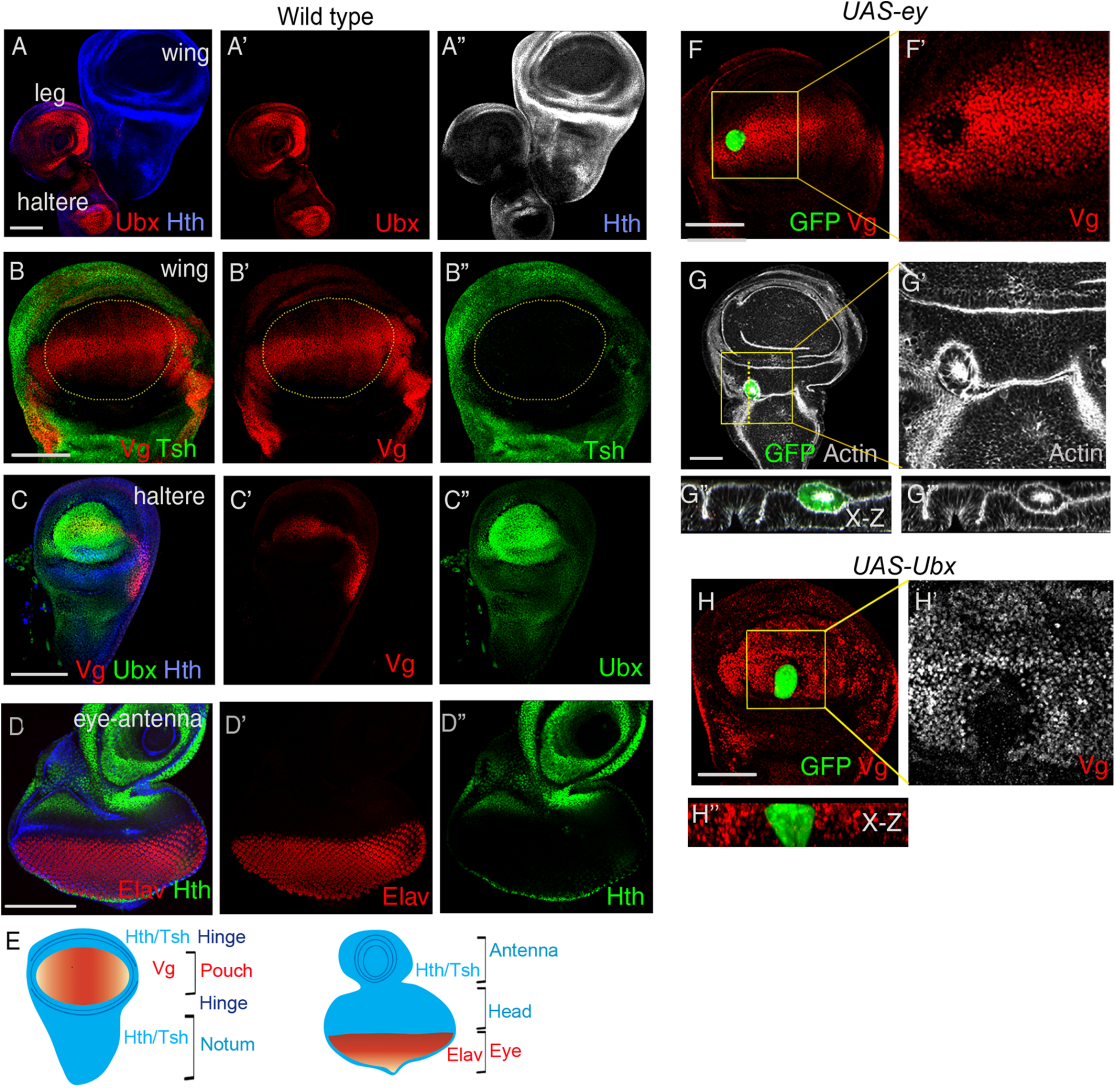
**Selector genes mark different** **developmental domains.** (A) Ubx (red) is expressed in the haltere and third leg disc, while it is absent in the wing disc. Hth (blue) marks the proximal domain. (B) Vg (red) is expressed in the distal wing pouch (marked by dotted line) while Tsh (green) is expressed by cells of the proximal wing. (C) Expression of Vg (red), Ubx (green) and Hth (blue) in the haltere. (D) Elav (red) is expressed in differentiated photoreceptors, while Hth (green) is expressed in cells of the head, antenna and eye margin. (E) Schematic representation of the different domains of the wing and eye imaginal discs. (F) Somatic clones with ectopic gain of Ey (*act>UAS-ey*, green) in the wing pouch results in loss of Vg (red). (G) Ey-expressing clones are displaced from the wing epithelium (also see x-z section, G″). (H) Somatic clones with ectopic gain of Ubx (*act>UAS-Ubx*, green) in the wing pouch exhibit loss of Vg (red). (F′,G′,H′) Magnifications of the respective boxed regions on the left. Scale bars: 100 µM.

The segment-selector, Ubx, is expressed in the haltere and in the third leg imaginal discs (Fig. 1A), while in the wing imaginal disc, its expression is restricted to only the overlaying peripodial cells (Fig. S1A). Expression of the field-selector, Vg, is found in the cells of the distal wing (pouch) imaginal disc (Fig. 1B) (Kim et al., 1996; Williams et al., 1991). Presumptive wing cells also express markers such as Nubbin (Fig. S1B) (Ng et al., 1995). On the other hand, cells outside the distal domain express Tsh (Fig. 1B; Fig. S1C) and/or Hth (Fig. S1B) (Azpiazu and Morata, 2000; Casares and Mann, 2000; Wu and Cohen, 2002). Along with Ubx, Vg also regulates haltere cell fate (Weatherbee et al., 1998; Williams et al., 1991) and is expressed in the presumptive capitellum of the haltere imaginal disc (Fig. 1C). In the eye imaginal disc, on the other hand, differentiating retinal cells express a neuronal marker, Elav (Fig. 1D) (Robinow and White, 1991), while cells outside the eye field express Hth (Fig. 1D) (Peng et al., 2009) and/or Tsh (Bessa et al., 2002). Ectopic gain of field selector, such as Ey, in another developmental domain, such as the wing imaginal discs, results in loss of wing cell fate, marked by loss of field selector Vg (Fig. 1F) and a distal wing cell fate marker Nub (Fig. S1D). Cells displaying altered cell fate sort out and are displaced from the epithelial plane as protruding polyps (Fig. 1G) (Bielmeier et al., 2016). Further, gain of Ey can result in ectopic eye formation (Halder et al., 1995) coincident with domain of expression of Tsh, as in the hinge region of the wing imaginal disc (Fig. S1E,F) (Bessa et al., 2002). Likewise, ectopic gain of Ubx reverses wing cell fate, as revealed by loss of expression of Vg (Fig. 1H) (Weatherbee et al., 1998) and concomitant gain of Tsh (Fig. S1G).

These larval imaginal discs with their well-defined segmental and field fates (Fig. 1E) thus offer ideal model organs to probe the developmental contexts of tumor cooperation by deregulated selectors.

### Ey and Ubx selectors are tumor drivers in the proximal wing epithelium

**Fig. 2.**
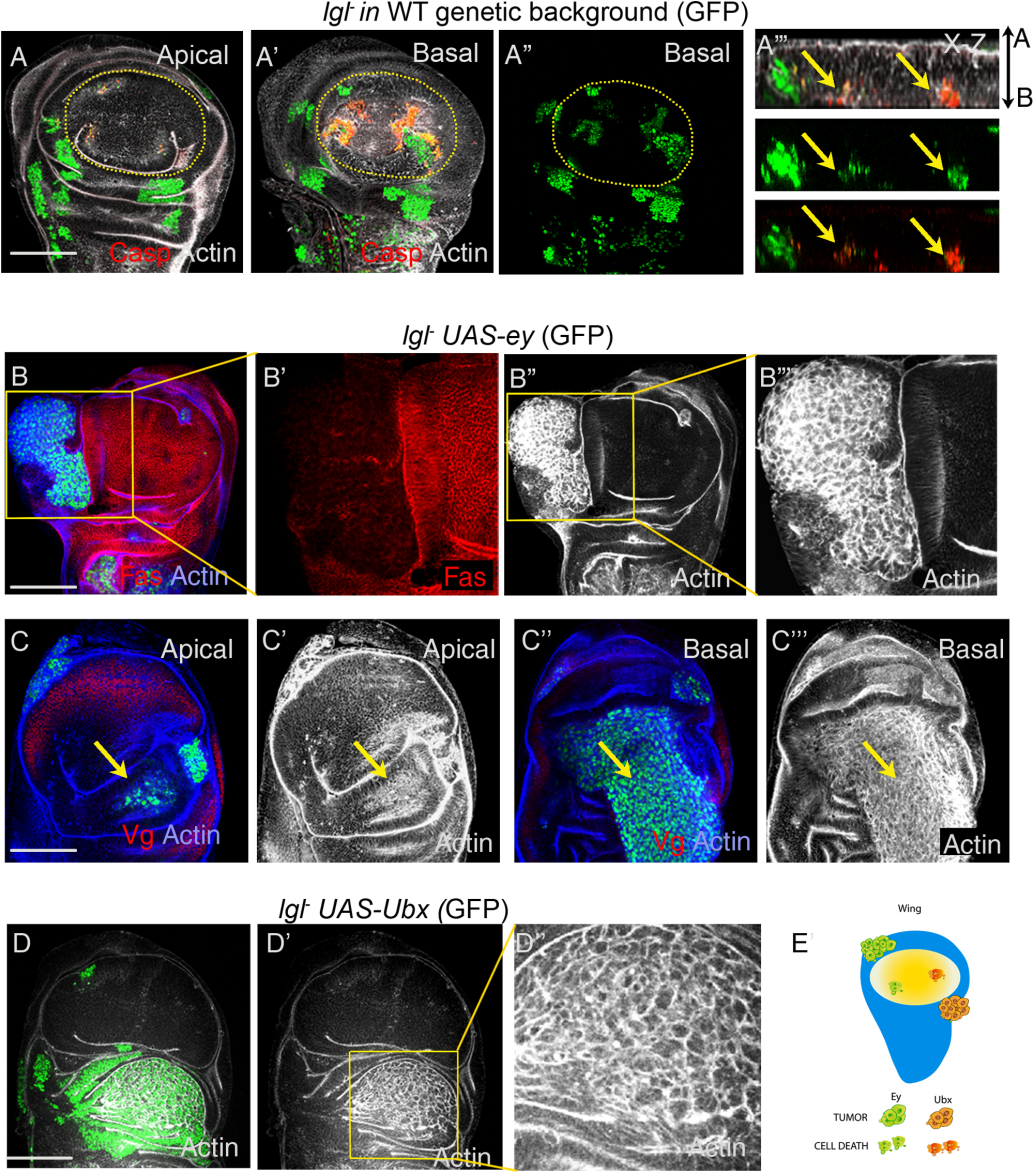
**Ey and Ubx drive neoplastic transformation of *lgl^−^* clones in the proximal wing.** (A) Cells with loss of Lgl (green) display cell death, as seen by gain of caspase (red), and undergo basal extrusion (see x-z section, A‴; in this and all subsequent x-z sections, A and B represent, respectively, the apical and the basal ends of the columnar epithelium). (B) *lgl^−^ UAS-ey* clone (green) displays neoplastic transformation (disrupted F-actin, grey) in the proximal wing, and exhibits loss of septate junction marker, FasIII (red). (C) A basally extruded *lgl^−^UAS-ey* tumor (green, arrow) in the proximal wing. (D) *lgl^−^ UAS-Ubx* clone (green) display neoplastic transformation in the proximal wing (F-actin, grey). (E) Schematic representation of selective tumorigenesis of *lgl^−^* cells by Ey and Ubx in the proximal wing. (B′,B‴,D″) Magnifications of the respective boxed regions on the left. Scale bars: 100 µM.

Oncogenically targeted cells in *Drosophila* imaginal epithelium, for instance, those displaying somatic loss of Lgl, are eliminated by cell competition (Agrawal et al., 1995; Froldi et al., 2010; Khan et al., 2013). *lgl*^−^ cells drop basally, display high level of caspase, and are finally extruded from the epithelium (Fig. 2A). However, these *lgl^−^* somatic clones transform neoplastically when these are induced amongst *Minute/+* heterozygous cells that are compromised for cell competition, or when provided with the advantage of cell proliferation and cell competition by a gain of Yki, a target of Hippo pathway (Khan et al., 2013; Menendez et al., 2010).

Previously, we reported that neoplastic transformation of *lgl^−^* clones in the wing pouch is preceded by loss of Vg (Khan et al., 2013), the wing fate selector. This raises the possibility that events leading to switch in developmental fates of oncogenically targeted cells could be tumor promoting. We thus hypothesized that the selector genes, Ey and Ubx, by virtue of their ability to reprogram the wing field (see Fig. 1F,H), are likely to cooperate for *lgl^−^* neoplasia in the wing epithelium. By using MARCM technique (Lee and Luo, 2001), we thus generated *lgl^−^* somatic clones displaying simultaneous loss of Lgl and gain of either Ey or Ubx in the wing imaginal discs, and assayed for their neoplastic transformations based on their altered cyto-architecture as revealed by disrupted F-actin filament organization (Froldi et al., 2010; Khan et al., 2013; Menendez et al., 2010). We observed that *lgl^−^* clones displaying gain of Ey (*lgl^−^UAS-ey,* Fig. 2B,C, *n*=11/16) or Ubx (*lgl^−^UAS-Ubx,* Fig. 2D, *n*=8/12) were not eliminated by cell competition, unlike their *lgl^−^*counterparts in wild-type genetic background (Fig. 2A) and, instead, displayed neoplastic transformation marked by their characteristic loss of F-actin architecture (Fig. 2B″,C‴,D″) as well as loss of the septate junctions marker, Fas-III (Fig. 2B′). These clones also sorted out from their neighbors as seen from their smooth clone boundary (Fig. 2B,D). All the neoplastically transformed *lgl^−^* clones, however, were seen in the Hth/Tsh expressing domain of the proximal wing (hinge or notum, Fig. 2B,D), while none were seen in the distal wing epithelium. Further, neoplastically transformed *lgl^−^* clones in the proximal wing, unlike their distal counterparts, did not display cell death (absence of caspase, see below), thereby suggesting that selector genes confer tumor cooperation largely by overriding elimination of *lgl^−^* cells by apoptosis. We further observed that proximally transformed *lgl^−^*clones were often extruded from the epithelial plane, either basally (Fig. 2C) or apically (Fig. S2A). Previously it was noted that neoplastically transformed *lgl^−^* clones were largely extruded apically (Tamori et al., 2016). Of note, neoplastically transformed and extruded *lgl^−^UAS-ey* or *lgl^−^UAS-Ubx* clones were distinctly larger as compared to the control clones, *UAS-ey* and *UAS-Ubx*, respectively, which too were displaced from the epithelial plane as smaller polyps (see Fig. 1G,H). Further, the extruded *lgl^−^* clones displayed complete loss of actin architecture, unlike the polyps formed by the control clones that retain their normal actin cytoarchitecture (Bielmeier et al., 2016). Finally, like Ubx, other Hox genes such as Dfd (Regulski et al., 1987), Scr (Struhl, 1982), Abd-A and Abd-B (Sanchez-Herrero et al., 1985) too cooperated in *lgl^−^* neoplasia in the proximal wing (Fig. S3A-D). The proximal Hth/Tsh-expressing domain of the wing imaginal disc thus behaves as a tumor hot spot (Fig. 2E) (Tamori et al., 2016).

### Distal wing is refractory to tumor cooperation by selector genes

**Fig. 3.**
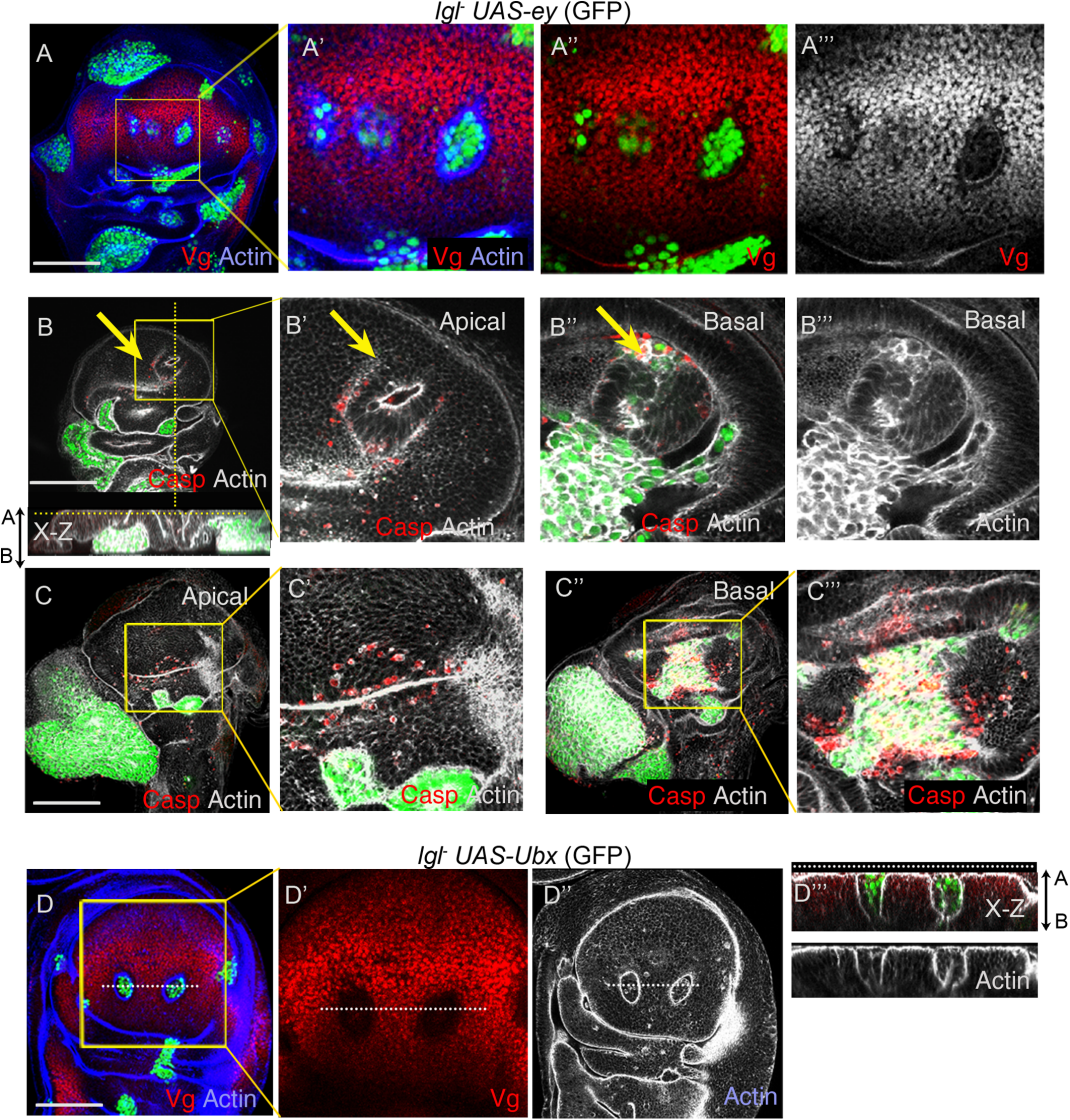
**Ey and Ubx fail to drive neoplastic transformation of *lgl^−^* clones in the distal wing.** (A,B) *lgl^−^ UAS-ey* (green) in the wing pouch (marked by Vg, red) display loss of Vg (red). Note that clones in the wing pouch are smaller than those in the proximal wing. (B,C) Distal clones show gain of caspase (red) and display extrusion (see characteristic actin cable, arrow); also see x-z section. Rare occurrence of neoplastic transformation (disrupted F-actin, grey) of an extruded clone (see basal sections, B″,C″). (D) *lgl^−^ UAS-Ubx* (green) clones (boxed area) in the distal wing display loss of Vg (red, D′) and display actin cable surrounding the clones (D″). (A′-A‴,B′-B‴,C′,C‴,D′) Magnifications of the respective boxed areas on the left. Scale bars: 100 µM.

Unlike the proximal wing, *lgl^−^ UAS-ey* and *lgl^−^ UAS-Ubx* clones display distinct characteristics in the distal wing imaginal disc. *lgl^−^ UAS-ey* clones in the distal wing (Fig. 3A-C), for instance, appeared distinctly smaller than their proximal counterparts (see Fig. 2B,D). Further, these exhibited loss of Vg (Fig. 3A‴), were basally extruded (Fig. 3B,C; Fig. S4A, *n*=13/16) and often displayed cell death (Fig. 3B′,C′). On rare instances (*n*=3/16), these basally extruded clones exhibited neoplastic transformation (see basal sections, Fig. 3B″,C″). Likewise, *lgl^−^ UAS-Ubx* clones in the distal wing (Fig. 3D) were smaller compared to their proximal counterparts (compare with Fig. 2D) and failed to transform (Fig. 3D′, *n*=15/15). These also displayed loss of Vg (Fig. 3D′) and sorted out from their neighboring cells (see x-z view, Fig. 3D‴). Thus, despite the ability of Ey and Ubx to reverse endogenous distal wing cell fate commitment, these *lgl^−^* clones exhibited poor growth and largely failed to display neoplastic transformation. Cell fate reversal therefore appears necessary, but not sufficient, for neoplastic transformation in the distal wing. Other factors such as local tissue and cell cytoarchitecture (Tamori et al., 2016) could possibly contribute to poor neoplastic propensity of the distal wing.

**Fig. 4.**
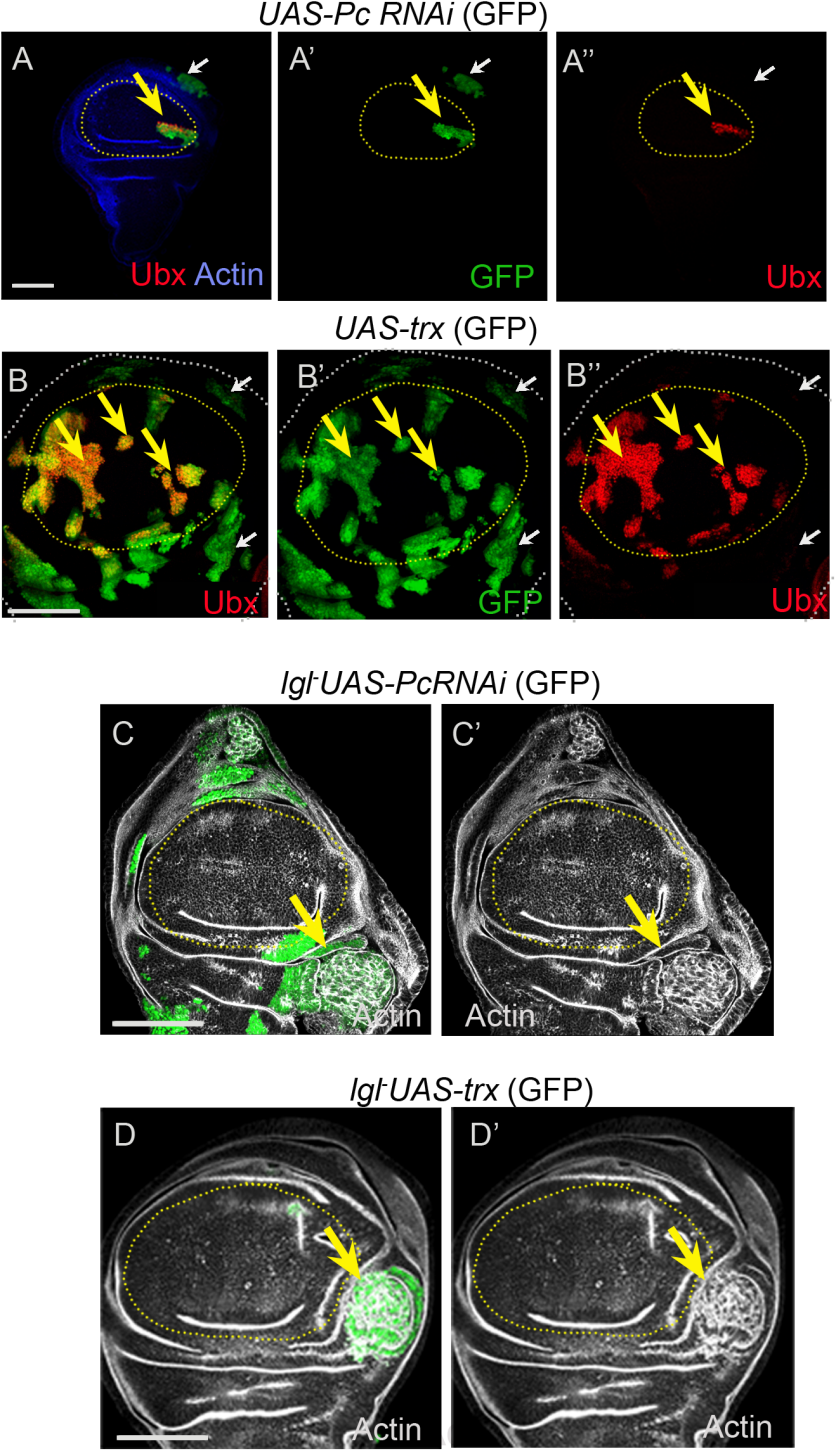
**Misexpression of epigenetic regulators drives *lgl^−^* neoplasia in only the proximal wing.** (A,B) Gain of Ubx in the wing pouch (yellow arrow) by loss of Pc (*act>UAS-PcRNAi,* green) or gain of Trx (*act>UAS-trx,* green). Note that most clones in the proximal wing (white arrows) do not display gain of Ubx. (C,D) *lgl* cells with loss of Pc (*lgl^−^ UAS-Pc RNAi,* green, arrow, C) or gain of Trx (*lgl^−^ UAS-trx,* green, arrow, D) undergo neoplastic transformation (disrupted F-actin, grey, C′,D′). Distal clones were not recovered in these samples, suggesting their early elimination. Scale bars: 100 µM.

Developmentally acquired or repressed cell fates are epigenetically maintained by the activities of the members of the PcG and TrxG complexes (Schwartz and Pirrotta, 2007). Loss of Polycomb, a member of the Polycomb Repressive Complex 1 (PRC1) (Beuchle et al., 2001), or gain of Trithorax (Trx), a global transcriptional activator, results in ectopic gain of Ubx in the distal wing (Fig. 4A,B, yellow arrows) (Klymenko and Müller, 2004; Sanchez-Elsner et al., 2006), and in few clones in the proximal domain of the wing imaginal disc (Fig. 4A,B). We thus sought to test whether *lgl*^−^ clones with loss of Pc (*lgl^−^UAS-Pc-RNAi,*) or gain of Trx (*lgl^−^ UAS-trx*) would recapitulate the consequence of gain of Ubx seen above (Fig. 3D). Indeed, as seen following direct gain of the Ubx (Fig. 3D), these Pc (Fig. 4C, *n*=7/11)- or Trx (Fig. 4D, *n*=6/9)-perturbed *lgl^−^*clones displayed neoplastic transformation in the proximal wing (Fig. 4C,D), while these were not recovered in the distal wing. However, it is likely that transformations of these clones in the proximal domains do not entail recruitment of Ubx and, instead, could be due to deregulation of other as yet unknown cooperative partners following loss of Pc or gain of Trx.

### Context-dependent tumor inhibition by Ey and Ubx drivers

**Fig. 5.**
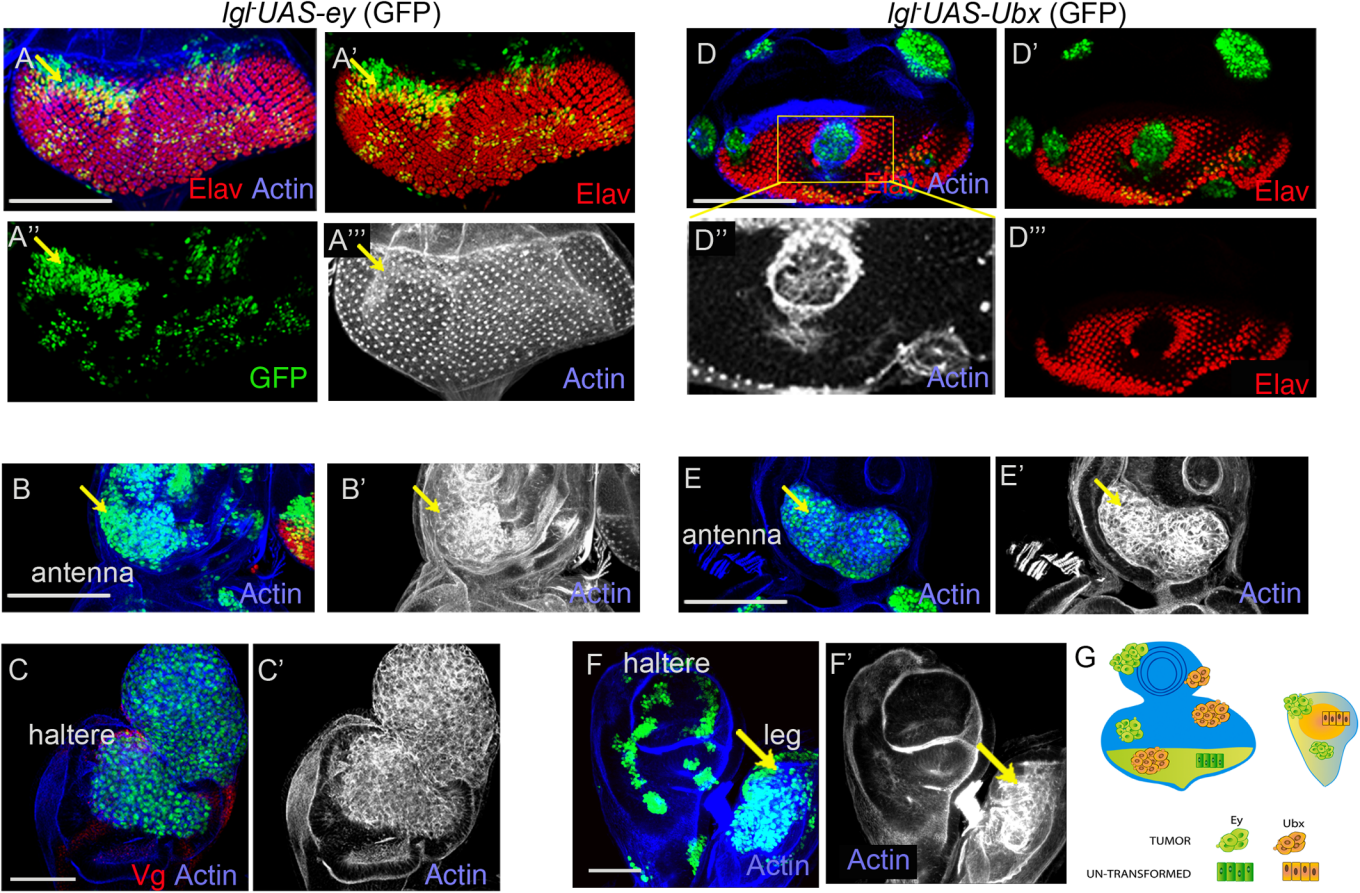
**Ey and Ubx fail to display *lgl^−^* neoplasia in their respective endogenous domains.** (A) *lgl^−^ UAS-ey* clones (green) do not undergo neoplastic transformation in the eye imaginal disc (intact F actin, grey, A‴), which is marked by Elav (red). These however display neoplastic transformation in the (B) antenna and (C) haltere epithelium (disrupted F-actin, grey). (D) *lgl^−^ UAS-Ubx* clones (green) in the eye disc display loss of retinal marker Elav (red) and show loss of cytoarchitecture (disrupted F-actin, grey, magnification of boxed region, D″), revealing neoplastic transformation. (E) *lgl^−^ UAS-Ubx* clones (green) in the antenna display neoplastic transformation (E′, actin); (F) however, in the haltere these are not neoplastically transformed (F′, actin). Note that these clones undergo neoplasia in the leg epithelium (F,F′, arrows). (G) Schematic representation of selective tumorigenesis of *lgl^−^* cells by Ey and Ubx in the eye and haltere imaginal discs. Scale bars: 100 µM.

Previously, we had shown that loss of Vg is essential for neoplastic transformation of *lgl* clones in the distal wing pouch (Khan et al., 2013). It was further seen that gain of Vg resulted in suppression of neoplasia in *lgl^−^*clones in both distal and proximal wing (Bunker et al., 2015; Khan et al., 2013). It is thus likely that the Vg selector gene behaves as a tumor inhibitor in the wing epithelium. By extension of this rationale, we further asked if Ubx and Ey too would inhibit neoplastic transformation of *lgl^−^* clones in their respective endogenous domains of expression. In the context of tumor cooperation of Ey selector in the eye disc, it should be noted that *lgl^−^* clones in the eye do not undergo elimination and instead retain their retinal cell fate (Grzeschik et al., 2007; Khan et al., 2013). We observed that *lgl^−^ UAS-ey* clones were indistinguishable (Fig. 5A) from those of the *lgl^−^* clones in the eye primordium. In other words, gain of Ey in *lgl^−^* clones in the eye primordium was inconsequential to the fate or development of the latter. Therefore, in this developmental domain, the Ey selector qualifies as a passenger mutation, meaning one that does affect tumor progression or elimination. By contrast, in the antenna (Fig. 5B, *n*=7/11) and haltere imaginal discs (Fig. 5C, *n*=5/9), these displayed neoplastic transformations.

On the other hand, *lgl^−^ UAS-Ubx* clones displayed neoplastic transformation in the eye primordium (Fig. 5D, *n*=9/15) with accompanying loss of the retinal fate marker, Elav (Fig. 5D‴). Such tumor cooperation by Ubx in the eye could be due to its ability to suppress retinal cell fate when ectopically expressed (Fig. S5A). Further, *lgl^−^ UAS-Ubx* clones also transformed in the Hth-expressing domain of the antennal disc (Fig. 5E, *n*=5/7). We noted that although *lgl^−^* clones (Fig. S5B) transform the haltere imaginal disc, those displaying gain of Ubx (*lgl^−^ UAS-Ubx*) failed to do so (*n*=21/21) despite their growth and survival (Fig. 5F). Together, these results reveal that Ey and Ubx selectors behave as tumor inhibitors in their respective endogenous domains (Fig. 5G).

The foregoing observations on the developmental domain-specific tumor driver or suppressor activity of selector genes also raise the possibility that Vg, which was previously seen to behave as a tumor inhibitor in the developing wing epithelium (Bunker et al., 2015; Khan et al., 2013), could behave differently in another developmental context. We note that flip out clones with ectopic gain of Vg failed to lose the retinal fate marker, Elav, in the eye disc proper (Fig. S6A). In agreement, we note that *lgl^−^* mutant clones displaying gain of the Vg selector (*lgl^−^ UAS-vg*) neither induced loss of retinal cell fate (Fig. S6B, arrowheads) nor facilitated neoplastic transformation in the eye disc (Fig. S6B, *n*=7/7) thus behaving as a passenger mutation in the eye. However, in the margin cells of the eye disc, which express Hth (see Fig. 1D″), *lgl^−^ UAS-vg* clones were transformed (Fig. S6B, yellow arrow). Thus, the Vg field selector qualifies as a driver mutation in the margin cells of the eye disc. Such roles as tumor driver by Vg were also observed in other developmental domains such as the leg epithelium, wherein *lgl^−^UAS-vg* clones underwent neoplastic transformation (Fig. S6C). Together, these results reveal that selector genes can be a tumor driver in one developmental domain while being a passenger or a tumor inhibitor in another.

### Tsh cooperates for *lgl^−^* neoplasia in multiple developmental domains

**Fig. 6.**
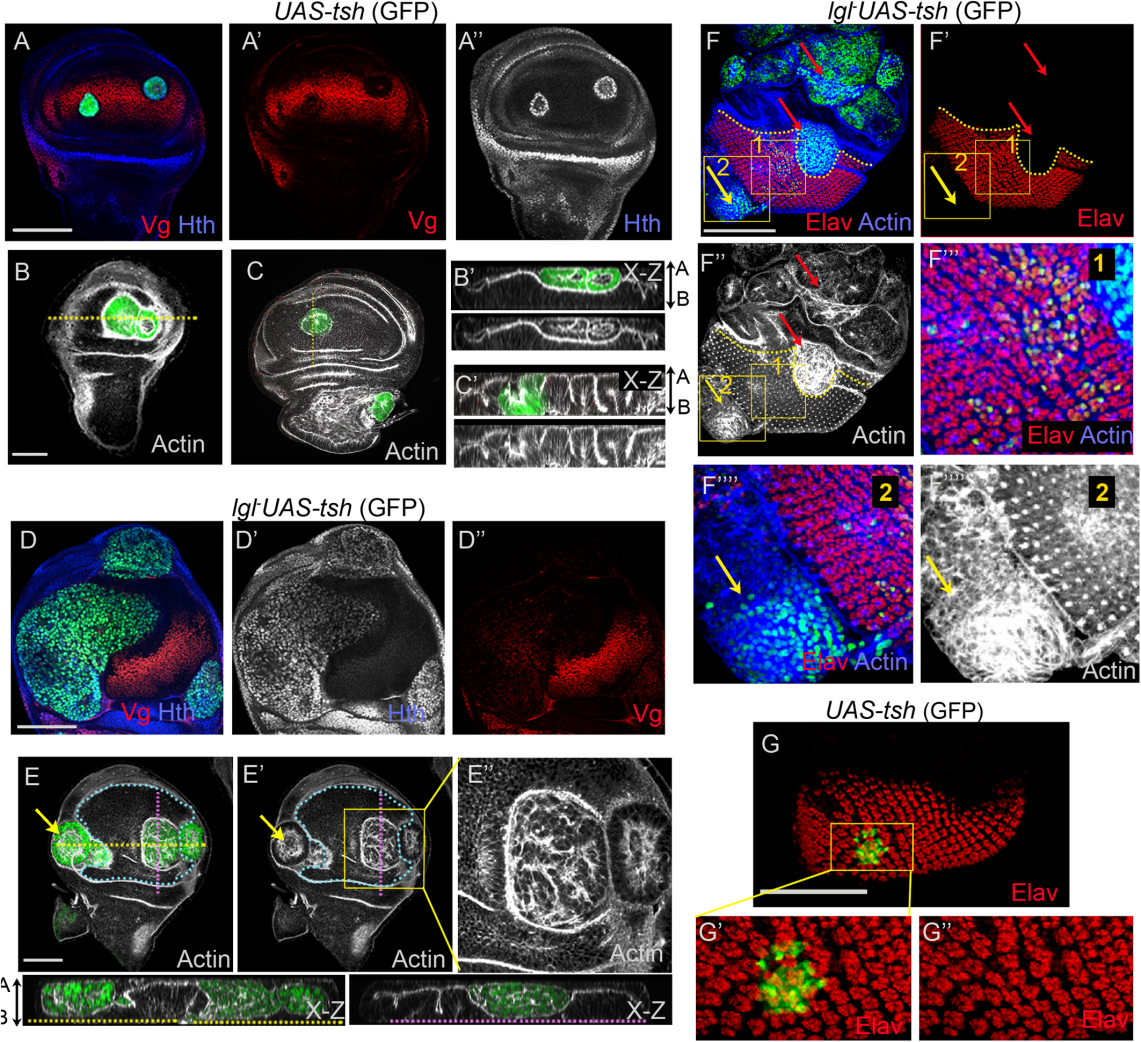
**Tsh drives *lgl^−^* neoplasia in multiple domains of wing and eye imaginal discs.** (A,B) Somatic clones with ectopic gain of Tsh (*act>UAS-tsh*, green) display loss of Vg (red, A′) and gain of Hth (blue, A″); these clones are extruded from the epithelial plane (see x-z section, B′,C′). (D) *lgl^−^ UAS-tsh* clones (green) display loss of Vg (D′) and concomitant gain of Hth (blue, D″) and undergo neoplastic transformation (disrupted F-actin, grey) in the wing pouch (marked by blue dotted line in E and E′) and in the proximal wing (arrows). x-z sections along the yellow and pink dotted lines in E are displayed in the panels at the bottom. (F) *lgl^−^ UAS-tsh* clones (green) fail to undergo neoplasia in the Elav expressing domain (boxed area 1), while in the eye margin these are neoplastically transformed (boxed area 2, yellow arrow), as seen from disrupted F-actin (grey). (G) Ectopic gain of Tsh (*UAS-tsh*, green) in the eye epithelium does not cause loss of Elav (red). (E″,F‴-F‴″,G′,G″) Magnifications of the respective boxed regions. Scale bars: 100 µM.

Ready neoplastic transformation of the *lgl^−^* clones by different selectors in the Hth/Tsh-expressing cells of the proximal wing (Fig. 2B-D) and antenna-head epithelia (Fig. 5B,E) suggest a permissive role of Tsh for tumor progression. By extension, we argued that gain of Tsh could also drive *lgl^−^* transformation in different developmental domains. It is known that ectopic gain of Tsh (*UAS-tsh*) in the distal wing results in reversal of wing fate, and a concomitant gain of proximal fate Hth (Fig. 6A) (Casares and Mann, 2000), often resulting in their extrusions, either apically (Fig. 6B) or basally (Fig. 6C). In agreement with these effects of gain of Tsh alone, we note that *lgl^−^ UAS-tsh* clones displayed neoplastic transformation both in the distal (Fig. 6D, *n*=5/11) and in the proximal (Fig. 6E, 13/15) wing imaginal disc. We noted that unlike *lgl^−^ UAS-ey* (Fig. 3A) and *lgl^−^ UAS-Ubx* clones (Fig. 3D), *lgl^−^ UAS-tsh* clones displayed higher incidence of transformation (*n*=5/11) in the distal wing pouch. Further, transformed *lgl^−^UAS-tsh* clones in the wing pouch were marked by loss of wing fate Vg (Fig. 6D), besides other distal cell fate markers, such as Dll (Fig. S7A) and sensory bristle marker, Cut (Fig. S7B), thereby, revealing reprogramming of the wing field. We observed that transformed *lgl^−^ UAS-tsh* clones were often seen extruded apically (Fig. 6E, see x-z sections), reminiscent of such behavior reported earlier (Tamori et al., 2016). We further ascertained that these apically extruded *lgl^−^ UAS-tsh* clones were not peripodial in origin, based on the absence of expression of a peripodial cell marker, Ubx (Fig. S7C) (Brower, 1987).Thus it could be speculated that tumor cold spots (Tamori et al., 2016), such as the distal wing, with their characteristic cytoarchitecture, are also dictated by their endogenous cell fate determinants. Such that upon loss of developmentally acquired cell fates such as Vg by ectopic gain of Tsh as seen here (Fig. 6), converts these cold spots to tumor hot spots.

*lgl^−^ UAS-tsh* clones, however, failed to undergo neoplastic transformation in the eye disc proper (Fig. 6F, box 1, *n*=11/11).This is consistent with the fact that gain of Tsh per se does not reverse eye cell fate (Fig. 6G) (Bessa et al., 2002) and, instead, can ectopically induce expression of proneural genes in the head and antenna (Bessa and Casares, 2005; Bessa et al., 2002; Pan and Rubin, 1998). We observed that *lgl^−^ UAS-tsh* clones, originating in the margin cells of the eye disc that express Hth, however, displayed neoplastic transformation (Fig. 6F, arrow, *n*=6/9); further, these were also transformed in the head domain of the eye disc (Fig. 6F, red arrow, *n*=7/13) and in the antennal disc (Fig. 6F, red arrow, *n*=9/14).

Together, these results reveal that Tsh cooperates for *lgl^−^* transformation both in its endogenous domains, and also when ectopically expressed. However, its ability to act as a driver mutation in ectopic domains is contingent on its ability to reverse cell fate commitment in the oncogenically targeted cell, as seen from its ability to induce *lgl^−^* neoplasia in the in the distal wing but not in the eye primordium.

## DISCUSSION

A large number of homeotic selector genes are deregulated in human cancers (Abate-Shen, 2002; Shah and Sukumar, 2010) and, yet, it is not clearly resolved if these follow certain ground-rules of their tumor cooperation or otherwise. Given that these selectors are critical for development, particularly early during organogenesis, their deregulation in tumors may mirror underpinnings of their essential developmental roles. Using the *Drosophila* model of epithelial carcinogenesis, we here have asked if such selectors display capacities for tumor cooperation and the developmental contexts thereof. The present study as well as earlier observations (Froldi et al., 2010; Khan et al., 2013; Tamori et al., 2016) have shown that tumorigenesis in larval epithelium displays spatial selectivity. Poor transformation of the distal wing domain could be overcome by manipulating different characteristic features of the distal wing, such as by loss of wing fate (Khan et al., 2013), or by disrupting tissue architecture (Bielmeier et al., 2016; Tamori et al., 2016) or levels of Myc (Froldi et al., 2010).We have chosen to test two selectors, Ubx and Ey, because their homologs are deregulated in human cancers, for their capacities to induced neoplastic transform of imaginal disc epithelial cells in cooperation with loss of the tumor suppressor, Lgl. Our results show that gain of Ey and Ubx cooperates to bring about neoplastic transformation of *lgl^−^* clones in only selected developmental domains of the eye-antennal, wing, haltere and leg imaginal discs, while in other domains these behave as tumor inhibitors, or as passenger mutations, in the last instance, their gains being inconsequential to the eventual fate of *lgl^−^* clones.

The Hth- and Tsh-expressing proximal wing (Froldi et al., 2010; Khan et al., 2013), in particular, the developing wing hinge (Tamori et al., 2016), has been shown to be a tumor hotspot, presumably due to endogenous activity of JAK-STAT (Ayala-Camargo et al., 2013). It may be recalled that Hth and Tsh expression are initiated early during development (Azpiazu and Morata, 2000; Casares and Mann, 2000; Wu and Cohen, 2002). Further, in the developing eye, Hth is known to maintain stemness of eye progenitor cells (Peng et al., 2009). Both Hth and Tsh are known to interact with the Ey and Ubx selectors during the normal course of appendage development. Ey, for instance, interacts with Hth to maintain a proliferative state of the eye progenitor cells (Bessa et al., 2002; Peng et al., 2009). Ubx, on the other hand, is known to interact with Hth to activate transcription of various tissue-specific genes (Choo et al., 2011; Slattery et al., 2011; Mann and Affolter, 1998). Recapitulation of such cross-talks of Ubx and Ey selectors with Hth and/or Tsh in *lgl^−^*clones may thus readily drive tumor progression in the proximal wing. In the distal wing or in the eye primordium, however, neoplasia requires reversal of terminal cell fate besides advantage of cell survival to override tissue surveillance (Khan et al., 2013) and cytoskeletal barriers to neoplasia (Tamori et al., 2016).

Our findings thus present a few underlying developmental underpinnings, which help predict if a selector gene, upon its gain in an oncogenically targeted cell, would behave as a driver, passenger or inhibitor of tumor progression. In its endogenous domain of expression, selectors behave as tumor inhibitors/passengers, while when expressed ectopically these could behave as a driver or a passenger. Vg selector, for instance, is a passenger in the wing primordium (Bunker et al., 2015; Khan et al., 2013), while being a driver in select domains of the eye margin cells or even in the leg. Likewise, Ubx is an inhibitor in the haltere while in the proximal wing it is driver. In the distal wing, however, *lgl^−^ UAS*-*Ubx* clones, despite reversal in cell fate, fail to provide growth advantage and are eliminated, like those of *lgl^−^* clones in wild-type genetic background. Ubx selector is thus a passenger in the distal wing. Following this paradigm, Ey selector too behaves as a passenger in its endogenous domain, the developing eye, since it fails to alter the fate of the *lgl^−^* clones therein. In the proximal wing, however, it is a driver.

**Fig. 7.**
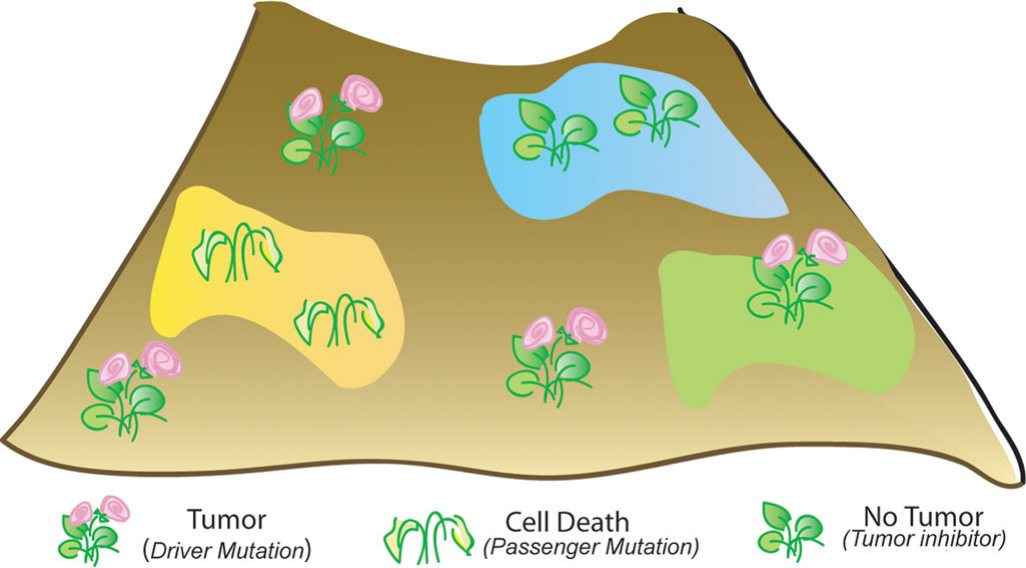
**Context-dependent tumor cooperation or inhibition by segmental and field selectors.** Conceptual rendition of *lgl^−^* clones (represented by plants) displaying gain of a selector gene (such as Ubx) in a landscape, the latter a metaphor for a developing organ primordium. The permissive/ground state of the landscape is marked in brown (e.g. Tsh-/Hth-expressing domains in the imaginal discs) while specialized areas are marked as colored patches (yellow, blue and green, equivalent to a developmentally specialized domain such as the eye, distal wing or haltere). The permissive/ground state readily allows growth and blooming of plants (tumor growth driven by selectors, e.g. *lgl^−^ UAS-Ubx* clones in proximal wing). In the specialized domain, plants either wilt (failure of Ubx to rescue elimination of *lgl^−^* clones, *lgl^−^ UAS-Ubx*, in distal wing) or grow but do not flower (rescue of *lgl^−^* clones by Ubx, *lgl^−^ UAS-Ubx*, from elimination in haltere). A developmental landscape therefore displays distinct propensities for neoplasia, due to developmental lineage and hierarchy, and thereby displays distinct outcomes upon gain of a given selector gene.

Taken together, our results provide a framework (Fig. 7) for tumor cooperation by selector genes and suggest a developmental underpinning of the hitherto complex pattern of association of deregulated selectors in diverse types of cancers (Shah and Sukumar, 2010). A particularly noteworthy feature of the present findings therefore is the distinct, and even opposing, fallouts of oncogenic gain of a given selector gene in different developmental contexts. A tumor-cooperating selector gene in one cell type could thus be a tumor inhibitor in another. In other words, it can be argued that it is the developmental lineage of an oncogenically targeted cell that determines its neoplastic propensities in the face of an oncogenic hit.

## MATERIALS AND METHODS

### *Drosophila* stocks

Fly stocks of the following genotypes were used in the present study: *lgl^4^* (#36289, Gateff, 1978), *UAS-ey* (#6294, Halder et al., 1995), *UAS-Ubx* (Castelli-Gair et al., 1994), *UAS-tsh* (Casares and Mann, 2000), *UAS-Pc-RNAi* (#33964, Ni et al., 2011), *UAS-abd-A* (#912, Greig and Akam, 1993), *UAS-Abd-B* (#913, Castelli-Gair et al., 1994), *UAS-Dfd* (#7299), *UAS-Scr* (#7302), *UAS-trx* (González and Busturia, 2009), *UAS-vg* (#37296, Kim et al., 1996), *Canton S.* and *yw; tub-Gal80 FRT40* (#5192) (# indicates Bloomington Drosophila Stock Center IDs). *UAS-tsh* was a gift from Richard Mann, Columbia University, *UAS-Ubx* a gift from L. S. Shasidhara, IISER Pune and *UAS-trx* a gift from Ana Busturia, Centro de Biología Molecular Severo Ochoa, Madrid.

### GFP-labeled somatic clone induction

GFP-labeled somatic clones were generated by mitotic recombination induced by giving heat-shock to larvae at 37°C for 30 min. Flip-out technique (Struhl and Basler, 1993) was used to generate transgene/RNAi-construct-expressing control clones; MARCM technique (Lee and Luo, 1999) was used to induce *lgl^−^* mutants with concomitant gain of selector gene function. Embryos were collected for a fixed time of 4 h on standard corn meal agar. Clones were induced 2 days after egg laying (AEL), and were analyzed 3 days after heat shock (AHS) for control, and 4 to 5 days AHS for test. Clones with loss of Lgl and gain of a cooperating partner, when they undergo neoplastic transformation, display extended larval life (Khan et al., 2013). These clones exhibit signs of neoplasia (disrupted F-Actin), starting 4 or 5 days after clone induction. On the other hand, larvae-bearing control clones undergo pupation by day 5 AEL; hence control clone-bearing larvae were dissected earlier, by day 3 AHS.

Detailed genotypes of the clones used in this study are listed in Table S1.

### Immunostaining

Primary antibodies: goat anti-Hth (1:100, Santa Cruz Biotechnology, dG-20, #sc-26187); rabbit anti-Tsh (1:500, Stephen Cohen, Department of Cellular and Molecular Medicine, University of Copenhagen, Denmark), rabbit anti-Vg (1:50, Sean Carroll, Howard Hughes Medical Institute, University of Wisconsin, Madison, USA), anti-mouse Nub (1:50, Steve Cohen). Secondary antibodies: Alexa Fluor dye-conjugated anti-goat, anti-mouse and anti-rabbit (Molecular Probes; A21428, A21070, A21422, A21082, A21424, A21094). Phalloidin-TRITC (Sigma-Aldrich, P1951).

### Image acquisition and analysis

Images were acquired with a Leica SP5 confocal microscope and processed using the Leica application software and Adobe Photoshop.

## Supplementary Material

Supplementary information
