# Supplementary material for: Selector genes display tumor cooperation and inhibition in Drosophila epithelium in a developmental context-dependent manner
Source: Biol Open. 2017 Nov 15;6(11):1581–91. doi: 10.1242/bio.027821 (PMC5703612; doi:10.1242/bio.027821)
Supplement: Supplementary information [file biolopen-6-027821-s1.pdf]

SUPPLEMENTARY DATA

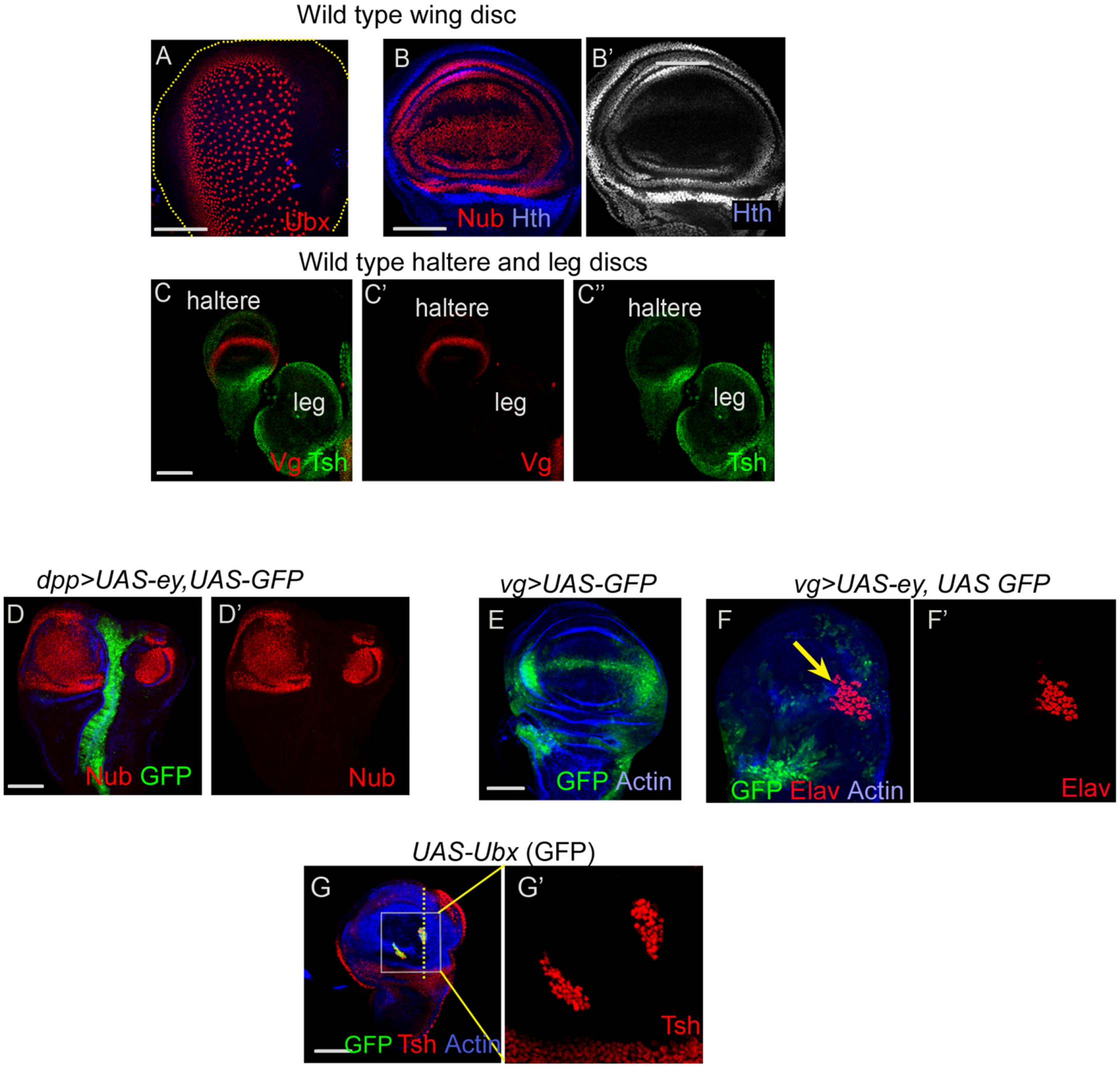

Fig.S1. Developmental domains of the wing imaginal disc and switch in their cell fates by ectopic gain of selectors. (A) Confocal projection of selected apical sections depicting expression of Ubx (red) in the overlying peripodial cells of the wing epithelium. (B) Nubbin (red) marks the distal wing domain while Hth (Blue) marks the proximal wing. Note that expression of Nubbin and Hth overlap at the hinge. (C) Expression of Tsh (green) and Vg (red) in the haltere and leg imaginal discs, Vg (red) is expressed in the haltere but not in the leg epithelium. (D) Loss of distal fate Nubbin (red) in cells expressing Ey, driven by *dpp-Gal4* driver (green). (E) Expression domain (green) of Vg driven by its boundary enhancer *vg-Gal4*. (F) Gain of neuronal marker Elav (red) in wing epithelium by ectopic gain of Ey (*vg-Gal4 > UAS-ey*, green). (G) Cells in wing pouch with somatic gain of Ubx (*act>UAS-Ubx*, green) results in ectopic gain of Tsh (red). Scale bars 100µM.

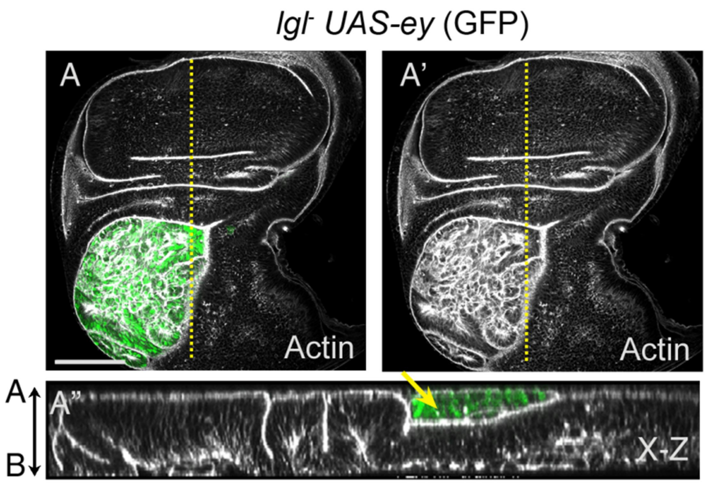

Fig. S2. Ey drives *lgl⁻* neoplasia in proximal wing (A) *lgl⁻* clones with gain of Ey (*lgl⁻ UAS ey,* green) undergo neoplastic transformation (disrupted F-actin, grey, A') and are extruded apically (see X-Z, A'', yellow arrow; in this and all subsequent X-Z sections, A and B represent, respectively, the apical and the basal ends of the columnar epithelium). Scale bar 100μM.

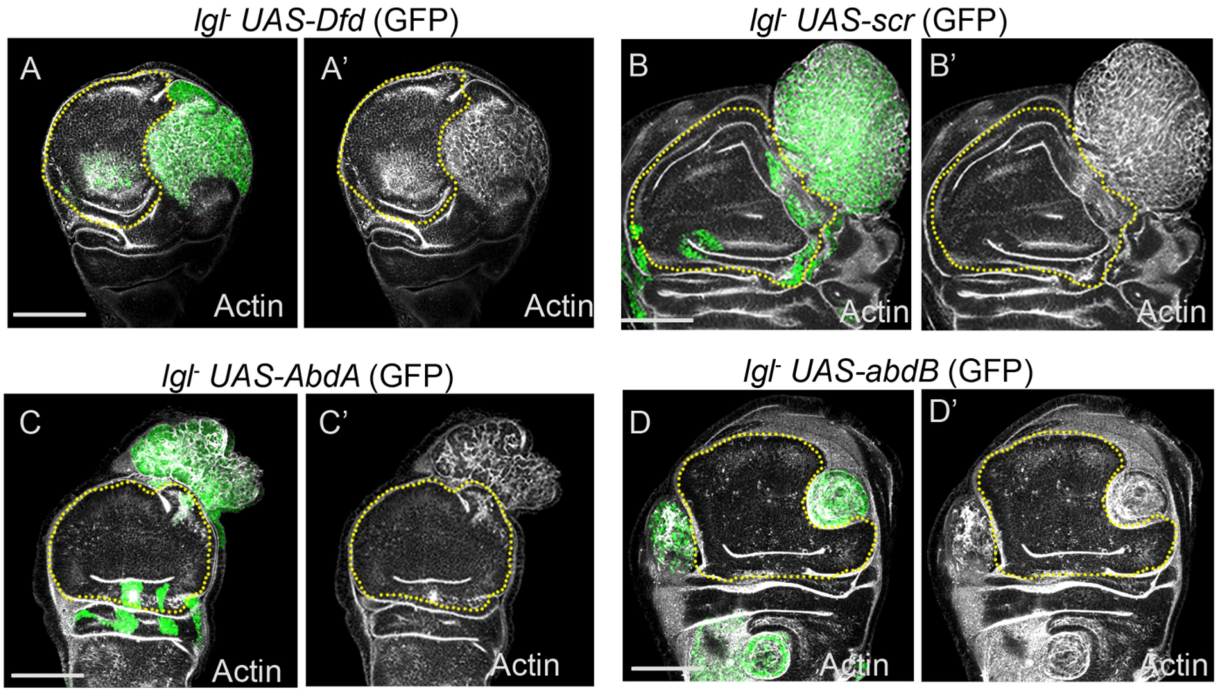

Fig. S3. Hox genes drive *lgl*⁻ neoplasia in proximal wing. (A-D) *lgl⁻* clones with gain of segment selectors (A) Dfd (*lgl⁻ UAS-Dfd,* green) (B) Scr (*lgl⁻ UAS-scr,* green) (C) Abd-A (*lgl⁻ UAS-Abd A,* green) (D) Abd-B (*lgl⁻ UAS-abd B,* green) undergo neoplastic transformation in the proximal wing (disrupted F-actin, grey, A', B',C', D'). Distal wing domain is marked by dotted line. Scale bars 100μM.

Biology Open • Supplementary information

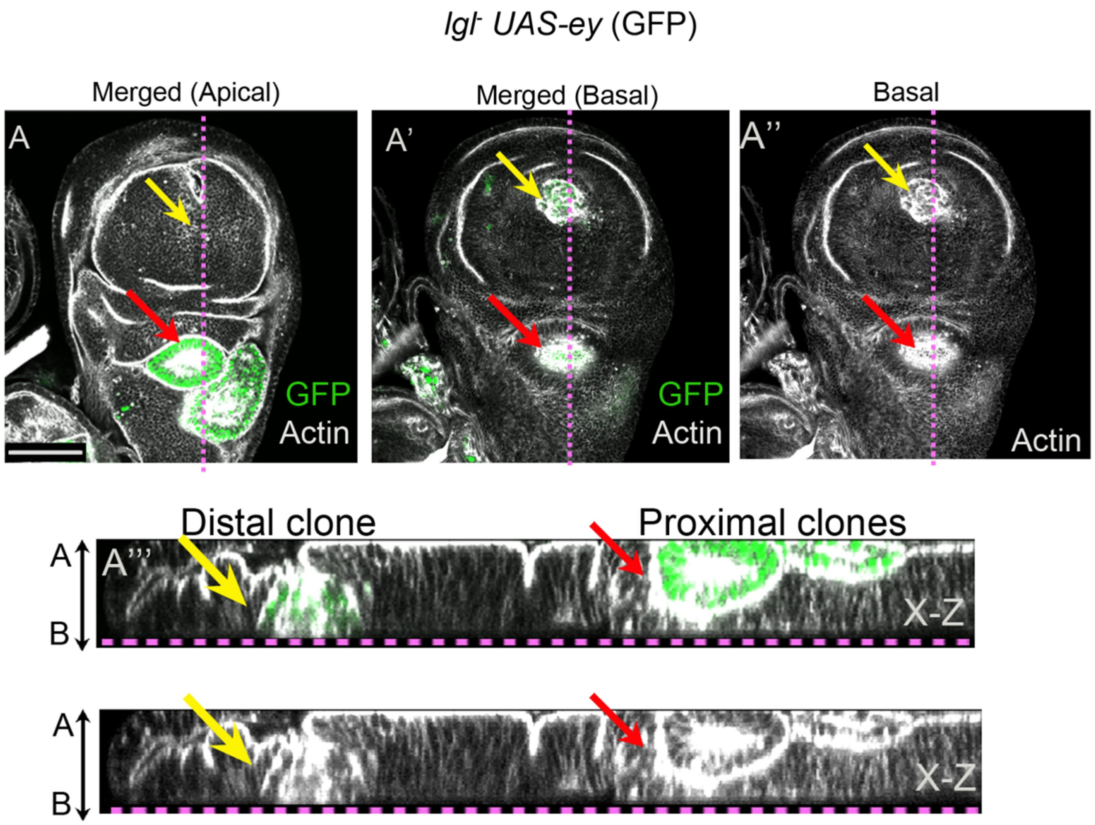

Fig. S4. *lgl-* clones displaying gain of Ey are extruded from the epithelial plane. (A) *lgl-* clones with gain of Ey (*lgl- UAS-ey,* green) are extruded from the wing epithelia either basally (yellow arrow, also see X-Z section A''') as seen in the distal wing pouch or apically (red arrow, also see X-Z section) as seen in the proximal wing. Scale bar 100µM.

Biology Open • Supplementary information

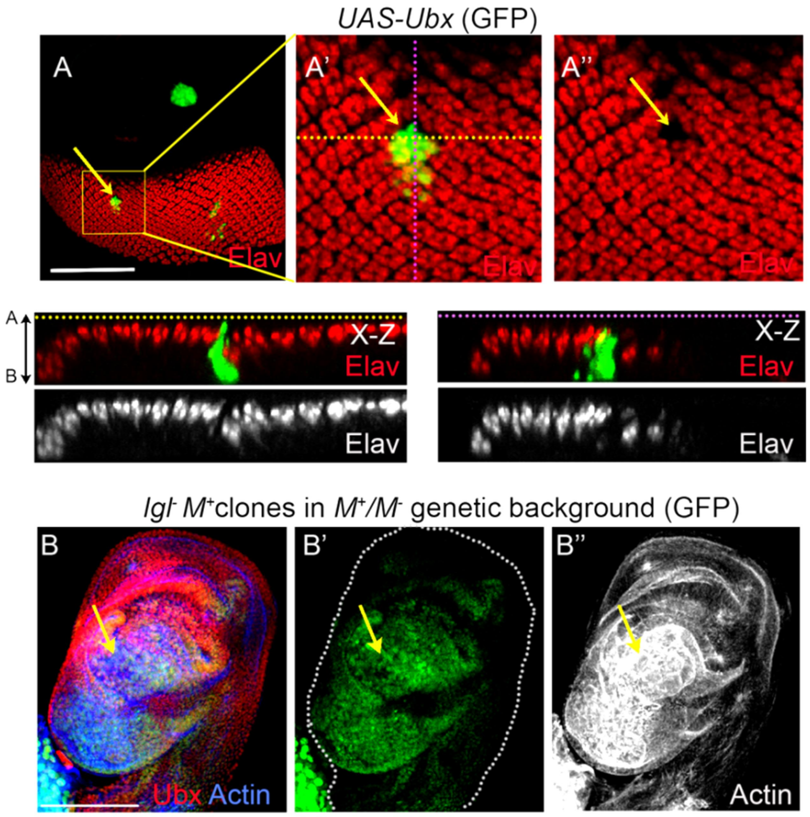

Fig. S5. Ectopic gain of Ubx results in loss of retinal marker Elav in the eye primordia. (A) Clones displaying gain of Ubx (*act*> *UAS-Ubx*, green) in the eye appear compromised for growth and show loss of Elav (red, arrow). Lower panels display the x-z sections across the dotted lines in A. (B) Neoplastic transformation of *lgl*⁻ clones in the haltere (marked by Ubx, Red) imaginal discs as seen by disrupted F-actin (grey). Scale bars 100µM.

Biology Open • Supplementary information

Biology Open • Supplementary information

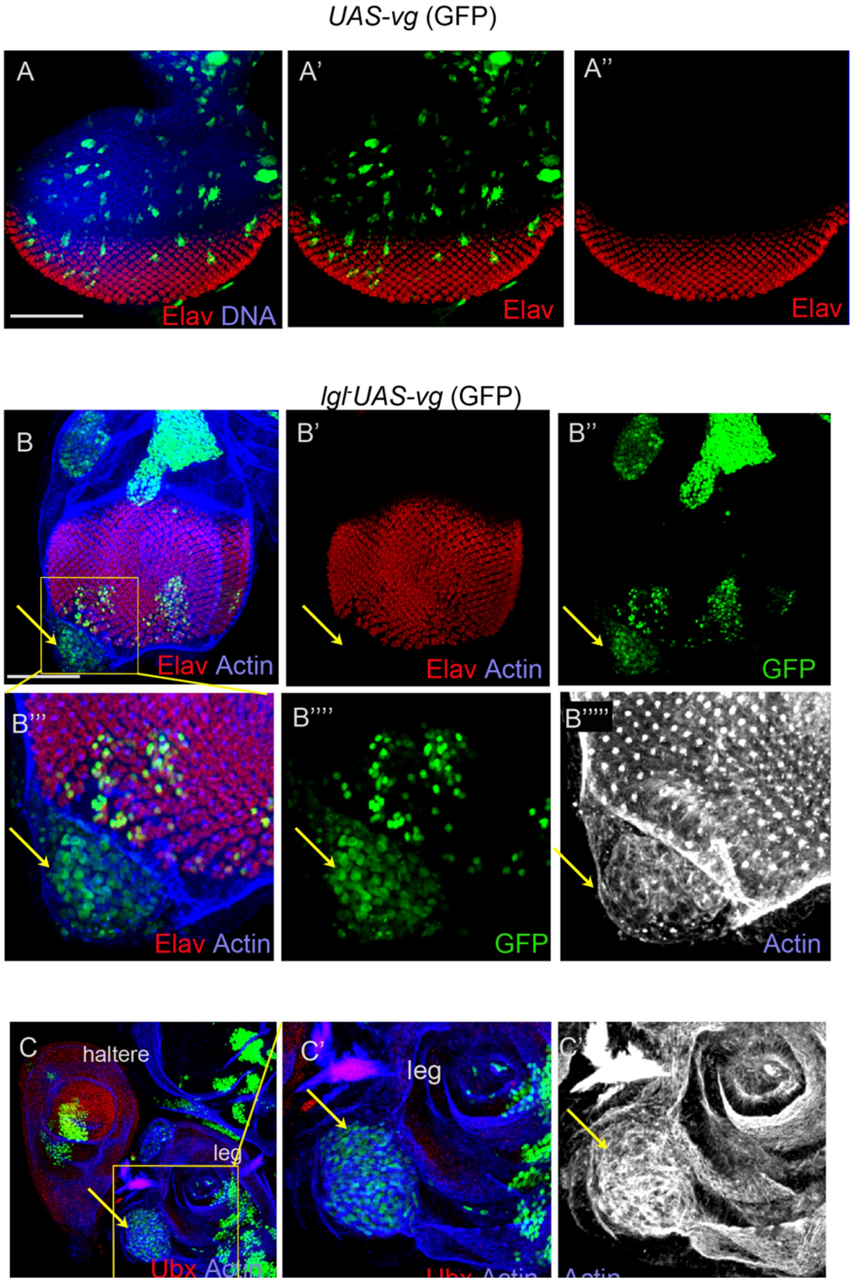

Fig. S6. Vg drives *lgl*$^-$ neoplasia in select developmental domains (A) Clones (GFP, green) display gain of Vg in the eye disc. (B-C) *lgl*$^-$ clones displaying gain of Vg (*lgl*$^-$ *UAS-vg*, green) in the eye disc. Boxed area in (B) is shown in the lower panel (B'''-B''''') to reveal neoplastic transformation of margin cells (arrow, F-actin, grey). (C) *lgl*$^-$ *UAS-vg* clones in leg imaginal disc (green, arrow) display neoplasia. Boxed area in (C) is displayed at a higher magnification (C'-C'') to reveal neoplastic transformation (F-actin, grey). Scale bars 100µM.

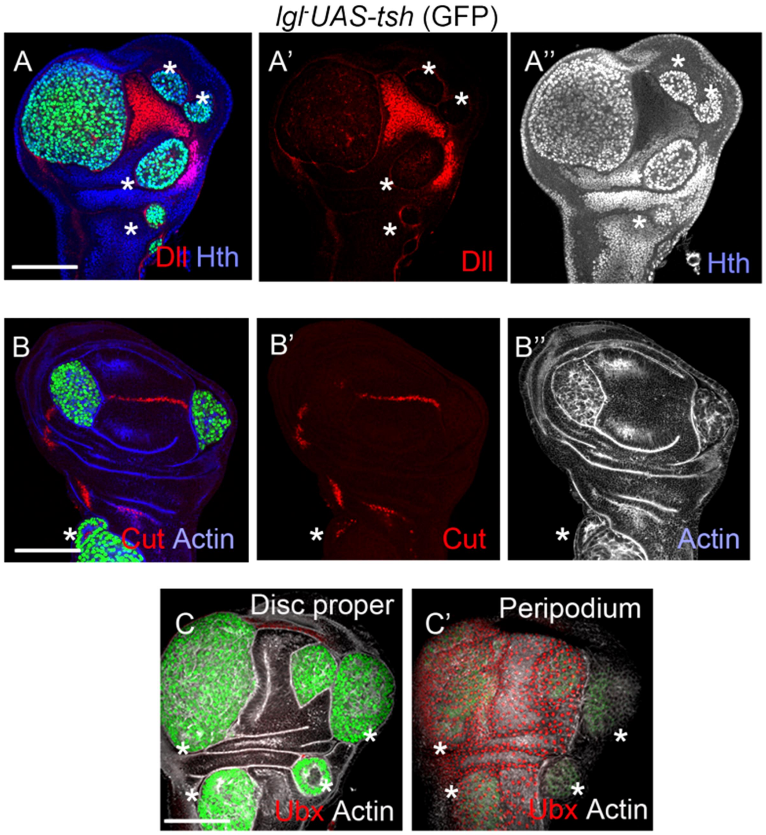

Fig. S7. Tsh drives *lgl⁻* neoplasia in distal wing and display fate reversal (A-D) *lgl⁻* clones with gain of Tsh (*lgl⁻ UAS-tsh*, green) display loss of distal fate as revealed by loss of (A) Dll (red) and (B) sensory marker Cut (red) and concomitant gain of proximal fate marker, Hth (blue, A''). Neoplastic transformation of these clones in distal wing is revealed by F-action (grey, B"). (C) Transformed *lgl⁻ UAS-tsh* clones (green) originated in the distal wing epithelium and not from the overlaying squamous peripodial cells—the latter group of cells are marked by large polyploidy cells that express Ubx (red). Proximal clones are marked by stars. Scale bars 100μM.

Biology Open • Supplementary information

Table S1: Detailed genotypes of the clones used in this study

Gain of selector genes
UAS-ey: *w hs-flp; act>y+>gal4, UAS-GFP/ UAS-ey*<br>***UAS-Ubx***: *w hs-flp; act>y+>gal4, UAS-GFP/UAS-Ubx*<br>***UAS-tsh***: *w hs-flp; act>y+>gal4, UAS-GFP/+; UAS-tsh/+*<br>***UAS-PcRNAi***: *w hs-flp; act>y+>gal4, UAS-GFP/+; UAS-PcRNAi/+*<br>***UAS-trx***: *w hs-flp; act>y+>gal4, UAS-GFP/+; UAS-trx/+*<br>***UAS-trx***: *w hs-flp; act>y+>gal4, UAS-GFP/+; UAS-vg/+*
lgl^- clones displaying gain of selector genes, epigenetic regulators or their loss
lgl^- UAS-ey: *y w hs-flp tub-gal4 UAS-GFP; lgl^- , UAS-ey FRT40/ tub-gal80 FRT40*<br>***lgl^- UAS-Ubx***: *y w hs-flp tub-gal4 UAS-GFP; lgl^- ,UAS-Ubx FRT40/ tub-gal80 FRT40*<br>***lgl^- UAS-tsh***: *y w hs-flp tub-gal4 UAS-GFP; lgl^- FRT40/tub-gal80 FRT40; UAS-tsh/+*<br>***lgl^- UAS-Dfd***: *y w hs-flp tub-gal4 UAS-GFP; lgl^- , UAS-Dfd FRT40/ tub-gal80 FRT40*<br>***lgl^- UAS-Scr***: *y w hs-flp tub-gal4 UAS-GFP; lgl^- , UAS-Scr FRT40/ tub-gal80 FRT40*<br>***lgl^- UAS-abd-A***: *y w hs-flp tub-gal4 UAS-GFP; lgl $^-$,UAS-abd-A FRT40/ tub-gal80 FRT40*<br>***lgl^- UAS-Abd-B***: *y w hs-flp tub-gal4 UAS-GFP; lgl^- , UAS-Abd-B FRT40/ tub-gal80 FRT40*<br>***lgl^- UAS-PcRNAi***: *y w hs-flp tub-gal4 UAS-GFP; lgl^- FRT40/ tub-gal80 FRT40; UAS-Pc-RNAi/+*<br>***lgl^- UAS-trx***: *y w hs-flp tub-gal4 UAS-GFP; lgl^- FRT40/ tub-gal80 FRT40; UAS-trx/+*<br>***lgl^- UAS-vg***: *y w hs-flp tub-gal4 UAS-GFP; lgl^- FRT40/ tub-gal80 FRT40; UAS-vg/+*
